# Microarray Expression Profile of Myricetin-Treated THP-1 Macrophages Exhibits Alterations in Atherosclerosis-Related Regulator Molecules and LXR/RXR Pathway

**DOI:** 10.3390/ijms24010278

**Published:** 2022-12-23

**Authors:** Etimad Huwait, Rehab Almassabi, Sanaa Almowallad, Salma Saddeek, Sajjad Karim, Gauthaman Kalamegam, Zeenat Mirza

**Affiliations:** 1 Biochemistry Department, Faculty of Sciences, King Abdulaziz University, Jeddah 21589, Saudi Arabia; 2 Cell Culture Lab, Experimental Biochemistry Unit, King Fahd Medical Research Centre, King Abdulaziz University, Jeddah 21589, Saudi Arabia; 3 Biochemistry Department, Faculty of Sciences, University of Tabuk, Tabuk 71491, Saudi Arabia; 4 Chemistry Department, Faculty of Sciences, University of Hafr Al Batin, Hafr Al Batin 39524, Saudi Arabia; 5 Department of Medical Laboratory Sciences, Faculty of Applied Medical Sciences, King Abdulaziz University, Jeddah 21589, Saudi Arabia; 6 Center of Excellence in Genomic Medicine Research, King Abdulaziz University, Jeddah 21589, Saudi Arabia; 7 Center for Transdisciplinary Research, Department of Pharmacology, Saveetha Institute of Medical and Technical Sciences, Saveetha Dental College and Hospital, Chennai 600077, India; 8 Pain and Palliative Care Medicine, RMD Specialties Hospital, RMD Academy for Health, Chennai 600069, India; 9 Pharmaceutical Division, Nibblen Life Sciences Private Limited, Chennai 600061, India; 10 King Fahd Medical Research Center, King Abdulaziz University, Jeddah 21589, Saudi Arabia

**Keywords:** atherosclerosis, gene expression, microarray, myricetin, LXR/RXR signaling pathway, scavenger receptors

## Abstract

Atherosclerosis is a chronic inflammation characterized by macrophage infiltration, lipid deposition, and arterial wall thickening. Prevention of atherosclerosis by nutraceuticals is gaining attention. Myricetin, a dietary flavonol, is claimed to possess anti-atherosclerosis properties. We studied myricetin’s effect on the atherosclerosis-associated molecular mechanism. Cytotoxicity and proliferation testing to check the viability of myricetin-treated THP-1 macrophages and monocyte migration study in the presence and absence of myricetin was performed. The whole transcriptome analysis was conducted using the Affymetrix microarray platform. The Partek genomics suite for detecting differentially expressed genes (DEGs) and ingenuity pathway analysis was used to identify canonical pathways. Cytotoxicity assays exhibited no significant toxicity in THP-1 macrophages treated with different myricetin concentrations (10–200 μM). Genome-wide expression profiling revealed 58 DEGs (53 upregulated and 5 downregulated) in myricetin-treated THP-1 macrophages. Pathway analysis revealed inhibition of LXR/RXR activation and angiogenesis inhibition by thrombospondin-1 and activated phagocytosis in myricetin-treated THP-1 macrophages. The cytotoxicity assay shows myricetin as a safe phytochemical. In vitro and in silico pathway studies on THP-1 macrophages showed that they can inhibit THP-1 monocyte migration and alter the cholesterol efflux mediated via LXR/RXR signaling. Therefore, myricetin could help in the prevention of cell infiltration in atherosclerotic plaque with reduced risk of stroke or brain damage.

## 1. Introduction

Coronary disorders, particularly atherosclerosis, are inflammatory diseases characterized by monocyte penetration and macrophage differentiation to enhance localized inflammation [1]. Atherosclerosis is defined as an inferior chronic inflammation of the arteries, strongly associated with cholesterol accumulation. Disease development is affected by the innate and adaptive functions of the immune system, with the pathogenesis regulated by specific macrophages that have crucial importance at multiple disease stages [2].

Macrophages play a central role in the development of atherosclerotic plaques. Classically activated M1 macrophages are involved in the initiation and establishment of inflammation, and alternatively activated or M2 macrophages are linked to the resolution of inflammation. The plasticity of macrophages makes them appealing therapeutic targets as they can reduce the development of and alleviate existing atherosclerosis [3]. Understanding macrophage biology can help us better understand varied biological processes and provide insights into new immunomodulation-based therapeutic strategies. In vitro studies commonly use monocytes as precursor cells due to the low yield and technical difficulty in isolating primary macrophages. Monocytic cell lines offer a limitless supply of macrophage precursors [4]. The human leukemia monocytic cell line, THP-1, is widely used to study monocyte/macrophage functions, mechanisms, signaling pathways, nutrient and drug transport, and as a model to investigate immune-modulating effects of compounds [5].

Several well-known synthetic medications that are commonly prescribed and can regulate atherosclerosis include antiplatelet medicines, anticoagulants, cholesterol-lowering medicines, and blood-pressure-lowering angiotensin-converting enzyme inhibitors. Statins (namely simvastatin, atorvastatin, and pravastatin, among others) are the most effective drugs for decreasing cholesterol, especially the low-density lipids (LDLs) in blood circulation, despite a risk of side effects such as liver toxicity and muscular pain [6]. Studies have shown that this problem can be overcome by using nutraceutical derivatives that possess the anti-atherosclerotic activity and therefore promote human health. Flavonoid-derived foods have shown anti-atherogenic effects through their positive effect on disease-related characteristics [7]. The hydroxyl-flavone class of flavonoids, such as myricetin, quercetin, and kaempferol, have demonstrated a variety of biological and therapeutic activities [8,9,10]. Myricetin (Myr) is functionally such as fisetin, luteolin, and quercetin [11] and is extracted from vegetables, including hot peppers, onions, and spinach [9], as well as berries and tea [12]. It is noteworthy that increased consumption of fruit and vegetables is closely associated with cardiovascular disease (CVD) prevention [13] owing to their high fiber content and anti-dyslipidemic, antioxidant, antineoplastic, and anti-inflammatory activities, therefore protecting from CVD progression [14,15,16]. Moreover, Myr prevents the aggregation of cholesterol in the macrophage foam cells [17], significantly inhibits macrophage activation and suppresses inflammation in vitro and in vivo [18]. 

However, the anti-atherogenic mechanism is not yet fully understood. Previous studies have exclusively focused on the effect of Myr on the gene expression in various cell lines exposed to one of the key atherogenesis stimuli such as oxidized low-density lipoprotein (ox-LDL) [17] and lipopolysaccharides (LPSs) [19]. It is crucial to study the direct effect of Myr on the macrophages’ early phase to screen the genetic changes after single exposure, an aspect not fully studied before. In this work, the protective activity of Myr was examined by studying the THP-1 macrophage viability and proliferation, followed by migration studies. Further gene array-based expression profiling of THP-1 human macrophages coupled with pathway analysis was carried out to further elucidate the mechanisms of Myr as an atherosclerotic preventive nutraceutical. 

## 2. Results

### 2.1. Myricetin Exhibited No Significant Change to the Viability and Proliferation of THP-1 Macrophages

The THP-1 macrophage viability was determined by the LDH cytotoxicity assay after 24 h of incubation with 10, 25, 50, 100, and 200 μM myricetin. The viability for treated macrophages relative to vehicle control was evaluated as the percentage of optical density reading. The viability percentage showed moderate changes compared to the control, but these changes in results are remarkably close to expected, as no significant differences were observed in the viability of myricetin-treated THP-1 macrophages for all experimental concentrations (Supplementary Figure S1A). The THP-1 macrophage proliferation was measured using the crystal violet test. The proliferation after using variable myricetin concentrations exhibited a slight difference compared to the vehicle control but was not statistically significant (Supplementary Figure S1B). All values are reported as the mean value percentage and the standard error of the mean. THP-1 monocytes demonstrated an increase in migration with Myr (25 μM) compared to the vehicle control. The overall percentage of THP-1 monocyte migration was 66.34%, whereas a 26.34% increase was seen compared to the control (Supplementary Figure S2). The cell culture images after the viability assay and proliferation experiments showed morphological changes (Supplementary Figure S3).

### 2.2. Gene Expression Profiling of Myricetin-Treated THP-1 Cells

**Figure 1:**
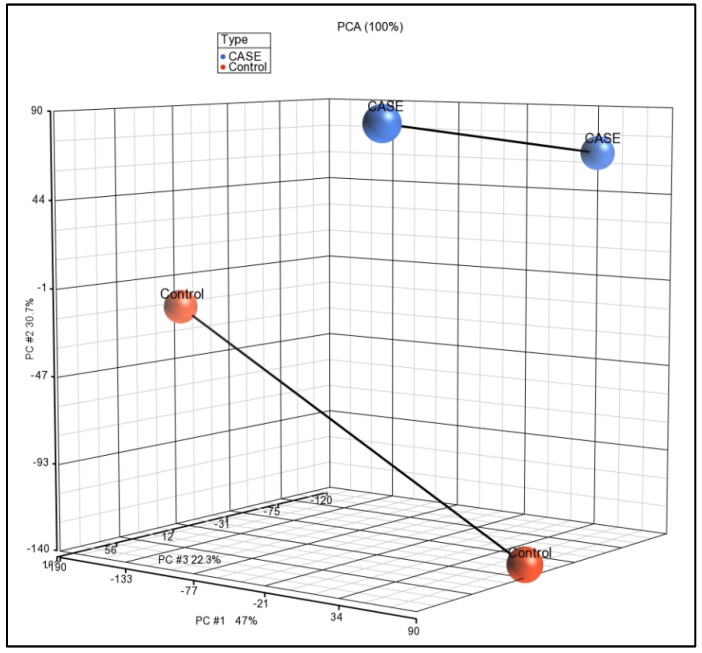
Scatter plot based on principal component analysis showing similarity of expression profiles of samples, indicated by dots. Blue and red colors indicate the case and control, respectively.

**Figure 2:**
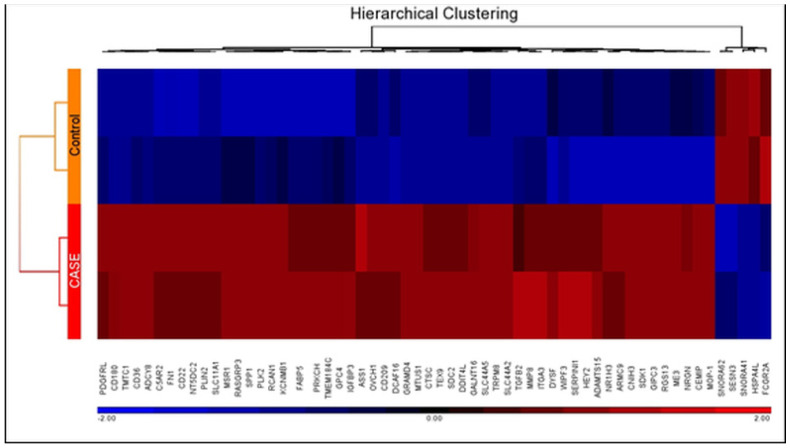
Hierarchical clustering and functional analysis of selected significantly differentially expressed genes in treated cell lines. Red color shows over-expression, and blue denotes under-expression in the heat map.

All the samples (cases and controls) were analyzed using Affymetrix Human ST 1.0 array and Partek GS 7.0 software. The expression data have been submitted to the NCBI’s GEO database with accession number GSE160430 (https://www.ncbi.nlm.nih.gov/geo/query/acc.cgi?acc=GSE160430 accessed on 20 June 2022). PCA scatter plot analysis for visualizing the high-dimensional array data, where each point represents a chip or sample, showed that the samples from the same type clustered together (Figure 1). FDR is an adjusted *p*-value to trim false positive results, and FDR offers more confidence than just the *p*-value. Usually, results below 0.05 are considered significant. We used experience-based FDR < 0.05 as a threshold and assumed the gene list has statistical significance. Comparison of the genome-wide expression of case and control groups by ANOVA revealed 58 statistically significant differentially expressed genes, amongst which 53 were over-expressed and 5 were under-expressed. The up-regulated genes included macrophage scavenger receptor 1 (*MSR1*), CD36 molecule as thrombospondin receptor (*CD36*), cell migration-inducing protein (*CEMIP*), microtubule-associated tumor suppressor 1 (*MTUS1*), fatty acid binding protein 5 (*FABP5*), fibronectin 1 (*FN1*), matrix metallopeptidase 8 (*MMP8*), nuclear receptor subfamily 1, group H member 3 (*NR1H3*), perilipin 2 (*PLIN2*), protein kinase C, eta (*PRKCH*) and secreted phosphoprotein 1 (*SPP1*) (Table 1, Figure 2). Down-regulated ones were small nucleolar RNA, H/ACA box 62 (*SNORA62*), Fc fragment of IgG, low-affinity IIa, receptor (*FCGR2A*), heat shock 70kDa protein 4-like (*HSPA4L*), sestrin 3 (*SESN3*), and small nucleolar RNA H/ACA box 41 (*SNORA41*).

### 2.3. Gene Ontology, Molecular Pathway, and Network Analysis 

**Figure 3:**
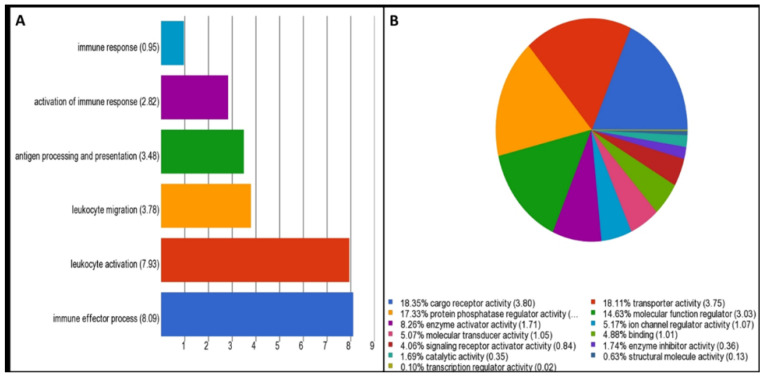
Gene set analysis result showing GO annotation for affected (**A**) biological process (immune effector (enrichment score 8.09)) and (**B**) molecular functions (cargo receptor activity (enrichment score 3.80)).

**Figure 4:**
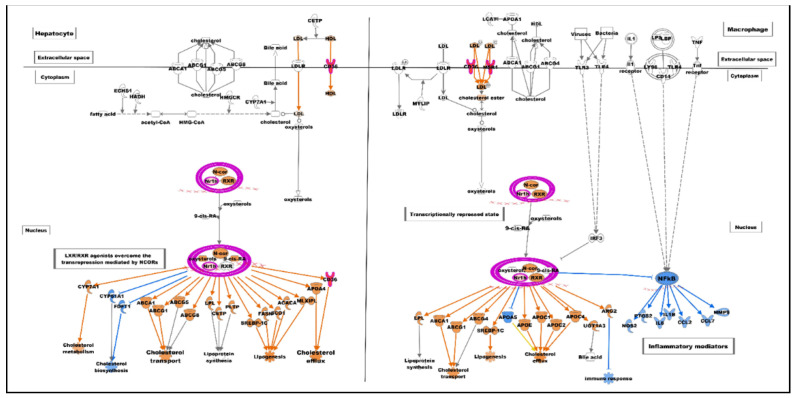
The ingenuity pathway analysis tool (IPA version 81348237) predicted significant activation of the LXR/RXR activation pathway (*p*-value = 3.06 × 10^−3^, Z-score = 2.3). The retinoid X receptors (RXRs) and liver X receptors (LXR) are nuclear receptors that mediate the biological effects of retinoids and livers, respectively. LXR regulates ABCA1 transported gene, which effluxes cholesterol from extrahepatic cells. Activated LXR/RXR pathway was overlaid with three differentially expressed genes [ratio = 0.024 (3/123), CD36, MSR1, NR1H3], which are associated with increased cholesterol metabolism, efflux, and transport and decreased cholesterol biosynthesis, immune response, and inflammatory mediators via inhibitory effect of NF-Kb pathway molecules. Red and orange colors indicated increase/activation, while blue color indicated decrease/inhibition.

**Figure 5:**
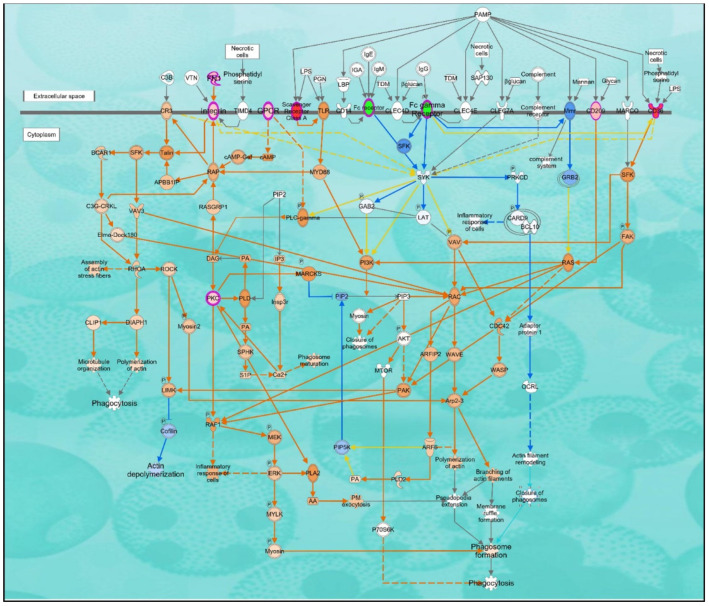
The ingenuity pathway analysis tool (IPA version 81348237) predicted significant activation of the phagosome formation pathway (*p*-value = 2.02 × 10^−4^, Z-score = 2.12). Multiple receptors (Fc, compliment, and scavengers’ receptors) simultaneously recognize the foreign particles/molecules/cells/microbes and initiate the phagosome formation. Activated phagosome formation pathway was overlaid with eight differentially expressed genes [ratio = 0.012 (8/691); CD36, MSR1, C5AR2, CD209, FCGR2A, FN1, ITGA3, PRKCH] and associated with increased polymerization of actin and phagocytosis. Red and orange colors indicate increase/activation while blue color indicates decrease/inhibition.

**Figure 6:**
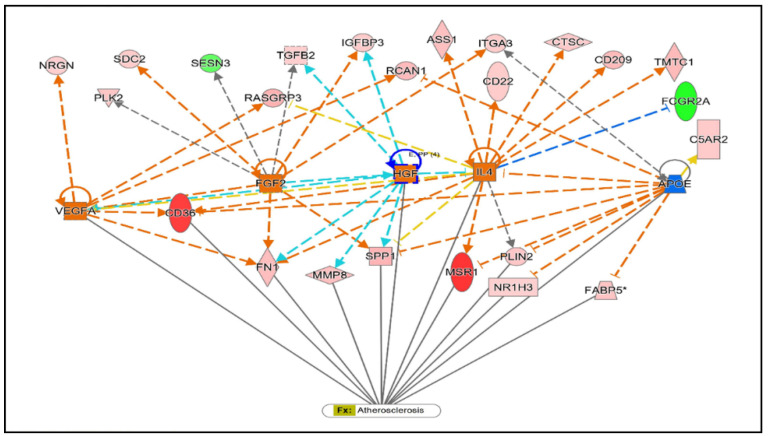
Predicted upstream regulators and their target genes in the network. The orange lines indicate activation, and the blue line represents the inhibitory effect. Disease and function overlay with target genes (*CD36*, *FN1*, *MMP8*, *SPP1*, *MSR1*, *NRIH3*, *FABP5*), and upstream regulators (*VEGFA*, *FGF2*, *HGF*, *IL4*, *APOE*) showing association with atherosclerosis.

Gene set analysis revealed immune effector (enrichment score 8.09) and cargo receptor activity (enrichment score 3.80) as the most significantly over-represented GO annotations in the categories of biological process and molecular function, respectively (Figure 3). The Ingenuity Pathway Analysis tool predicted the altered canonical pathways, upstream regulators, associated disease, physiological functions, networks, and cytotoxicity for uploaded DEGs. The most significant and disease-associated canonical pathways were LXR/RXR activation (Z-score = 2.31), VDR/RXR activation (Z-score = 2.13), inhibition of angiogenesis by thrombospondin-1 (TSP1) (Z-score = 2.21), and Phagosome Formation (Z-score = 2.12) (Table 2, Figure 4 and Figure 5). Cardiac Hypertrophy pathways were also found to be enhanced. Predicted upstream regulators were *VEGFA, FGF2, IL4, HGF, APOE,* and lipopolysaccharides. The network of upstream regulators and genes (*ITFA3, MSR1, SDC2, IGFBP3, CD209, TGFB2, DYSF, FCGR2A, SPP1, CD36, FN1,* and *PLIN2*) play a significant role in atherosclerosis by controlling the binding of epithelial cells, cell spreading, adhesion of immune cells, homing of cells and fatty acid metabolism (Figure 6).

### 2.4. Validation of the DNA Microarray by Using Real-Time Quantitative Polymerase Chain Reaction (q-PCR) 

**Figure 7:**
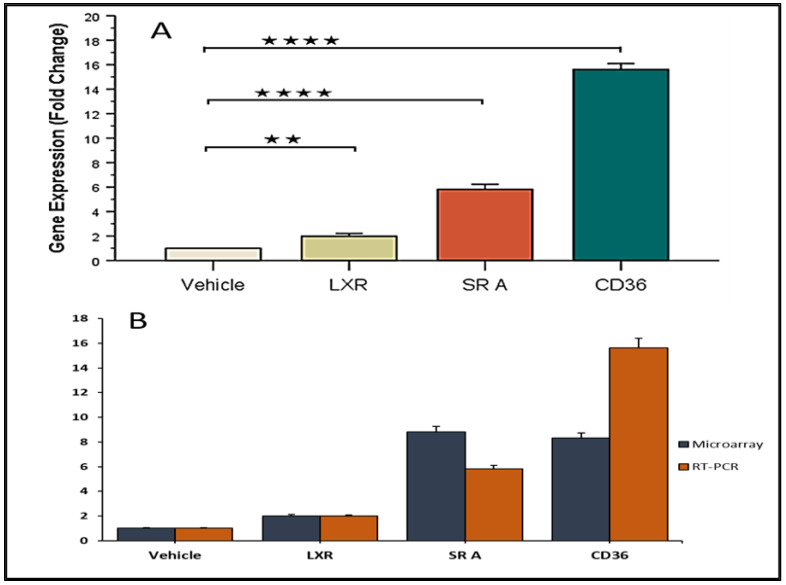
The gene expression of *LXR-α, SR-A,* and *CD36*; (**A**) qPCR result showing significant upregulation of *LXR-α, SR-A,* and *CD36* in myricetin-treated THP-1 macrophages. ٭٭ and ٭٭٭٭ indicate the statistical significance of *p*-value ≤ 0.01, and 0.0001, respectively, (**B**) Expression patterns of *LXR-α, SR-A,* and *CD36* in qPCR and microarray showing consistency.

The microarray data were validated by q-PCR. For analysis, target genes (*LXR-α, SR-A, CD36*) were normalized to the reference gene (*GAPDH*). The results showed significantly increased expression of target genes (*LXR-α* (*p*-value = 0.004, FC 2.1), *SR-A* (*p*-value = 0.001, FC = 5.9), *CD36* (*p*-value = 0.0001, FC = 15.7) in the THP-1 macrophages after treatment with Myr 25 μM for 24 h when compared to vehicle (Figure 7A). Additionally, the comparative study qPCR study and microarray showed similar expression patterns of target genes (Figure 7B).

## 3. Discussion

Atherosclerosis is a highly complex chronic inflammatory process with lipid and fibrous lesions of the vascular endothelium that create atherosclerotic plaque. In recent times, nutraceuticals have drawn attention as an alternative therapy for the prevention/treatment of atherosclerosis. Myricetin, a common flavonoid, has shown anti-diabetic, antioxidant, anti-cancer, and anti-inflammatory properties [12]. Myricetin is a well-known multifunctional glycoside molecule with multiple functional hydroxyl groups that can freely participate in its binding [20]. However, the therapeutic recommendation of myricetin requires the cytotoxicity assay to be passed without adverse effects. Myricetin exhibited “no cytotoxicity” as we found similar viability and proliferation results of myricetin-treated THP-1 macrophages and vehicle THP-1 macrophages. Our findings are in accordance with Cho et al., showing no cytotoxicity in RAW264.7 macrophages treated with different concentrations of myricetin (1 to 200 μM) by using the EZ-Cytox cell viability assay kit [16]. The protective effects of macrophages may be due to their inherent biological properties and may also be attributed to the rich presence of hydroxyl groups in myricetin’s chemical structure [21]. Cell migration studies in the presence and absence of myricetin support our hypothesis that myricetin may potentially aid in the prevention of cell infiltration.

Response to intracellular and extracellular signals plays a crucial role in regulating gene expression and transcription factors such as liver X receptors (LXR) in physiological and pathological environments of disease [22,23]. The LXRα, encoded by NR1H3, has an essential function in lipid metabolism and inflammatory processes [24]. The over-expression of the LXR-α gene (FC = 6.67 and *p*-value = 0.024) was detected in myricetin-treated cells, which might be an indication of cellular response to extracellular signals. 

The macrophages are key cells in the innate immune system that bind to various ligands through scavenger receptors [25]. The significant predicted alteration in the atherosclerosis-related LXR/RXR activation pathway (–log(*p*-value) = 4.62, Z-score 2.3), including two upregulated scavenger receptors, MSR1 and CD36, also known as scavenger receptor A1 (SR-A1) and scavenger receptor B2 (SR-B2), respectively, regulates a range of pathways influencing both atherosclerosis initiation and progression [25]. CD36 expression has been reported in diverse cell types, such as monocytes and macrophages, and it identifies various ligands (ox-LDL, FA, and TSP1) as well [26]. Over-expressed CD36 through TSP1 may induce the apoptosis signaling pathway and contribute to many biological processes such as cellular inflammation, adhesion, and migration [27], therefore inhibiting angiogenesis effectively in vitro and in vivo [28,29,30]. These explanations are well aligned with our finding for canonical pathways of inhibition of angiogenesis via the TSP1 pathway (–log (*p*-value = 2.5). Moreover, it is evident that CD36 expression could be affected by a change in hyperglycemic conditions [31,32]. Hyperglycemia is considered an important stimulator of CD36 mRNA synthesis in vitro [33]. Our results agreed with these studies in which the microenvironment of macrophages had a similar effect with 25 micrograms of myricetin. Moreover, macrophage apoptosis may be activated by the endoplasmic reticulum stress pathway with pattern recognition receptors that contribute strongly to the innate immune mechanism [34,35]. The ER stress pathway is triggered by ligands such as oxidation stress or ox-LDL to stimulate protein kinase (c-Jun N-terminal Kinase) [34].

Myricetin has a dual effect on the CD36 cellular microenvironment; increased CD36 expression in the absence of ox-LDL and decreased CD36 expression in RAW264.7 macrophages in the presence of ox-LDL [36]. Previously, the anti-angiogenic function of myricetin through apoptosis activation in HUVECs was shown [37]. Our results cast a strong light on the genetic anti-lipidemic effect of myricetin on cholesterol-free macrophages. One of the canonical pathways found during IPA analyses was cardiac hypertrophy, which reportedly is associated with coronary atherosclerosis. Key genes involved include ADCY8, ITGA3, PRKCH, RCAN1 and TGFB2. The nuclear factor of activated T cells (NFAT), along with calcineurin, also mediates the pathogenesis of cardiac hypertrophy and atherosclerosis [38,39]. Hence, Myr seems a potentially safe phytochemical capable of supplementing conventional therapies.

Though it is widely assumed that changes in specific mRNA levels are always accompanied by commensurate changes in the encoded proteins and vice versa, biology is full of exceptions and variations. In certain cases, mRNA expression might fail to accurately reflect protein abundance. Non-correspondence of mRNA and protein levels has been previously discussed [40,41,42]. Our study hypothesis and conclusion do suffer from this limitation. However, we would like to highlight that NR1H3 (Oxysterol receptor LXR-α; Nuclear receptor), SR-A, and CD36 were upregulated in the qPCR study. A similar profile was also evident from our microarray studies, and these reports from two independent platforms could be significant. Protein expression studies could not be performed as planned, and we consider this to be a limitation. However, we did observe similar expression profiles in both qPCR and the microarray, which suggests possible further effective translation and functional significance.

## 4. Materials and Methods

### 4.1. Chemical Materials and Culture Techniques 

The Human THP-1 cell line (TIB202) was kindly gifted by the Molecular Biomedicine unit of the King Faisal Specialist Hospital and Research Centre, Riyadh, KSA. RPMI-1640 media (A1049101), heat-inactivated fetal calf serum (FCS, A3840002), L-glutamine and 1% antibiotic solution (15140122, penicillin-streptomycin), dimethyl sulfoxide (DMSO) (D12345), the Pierce LDH cytotoxicity assay kit (8895) and phosphate-buffered saline (PBS 10010015) were purchased from Invitrogen (Invitrogen, Waltham, MA, USA). Phorbol-12-myristate-13-acetate (PMA, J63916) was purchased from Alfa Aesar (Thermo Fisher Scientific, Australia). Myricetin (70050, Sigma-Aldrich Company, Gillingham, UK) was dissolved in DMSO with a stock concentration of 1 mM. Complete RPMI-1640 medium supplemented with 10% HI-FCS, 2 mmol/L L-glutamine, and 1% penicillin-streptomycin was used to culture THP-1 cell lines in a humidified incubator (5% CO_2_) at 37 °C. Macrophage differentiation was achieved by incubating THP-1 cell lines with PMA (160 nM) at 37 °C for 24 h. For cell seeding, the supernatant of cultured cells was separated by centrifugation at 200× *g* for 5 min at room temperature and discarded, while the pellet was re-suspended in 1 mL complete medium, and cell density was evaluated using a hemocytometer.

### 4.2. Cell Viability and Proliferation Assay

The lactate dehydrogenase (LDH) enzyme frequently released during cell membrane injury was determined using the Pierce LDH cytotoxicity assay kit that reflects the cell viability. Differentiated THP-1 macrophages were seeded at a density of (4.11 × 10^5^) cells/cm^2^ in a 96-well plate with 100 μL of complete RPMI-1640 media. Cells were treated with various concentrations of myricetin (10, 25, 50, 100, 200 μM) or vehicle and incubated for an extra 24 h at 37 °C in a 5% CO_2_ incubator. PMA was not removed from the media. The supernatants and pellet were used the next day for the cell viability and proliferation assay. The supernatants (100 μL) were pipetted into a new 96-well plate and incubated with 100 μL of assay buffer at 37 °C for 30 min followed by 100 μL of stop solution to stop the reaction. The adherent macrophages were used in parallel for the proliferation assay by incubating 100 μL of crystal violet stain (0.2% (*w*/*v*) in 10% ethanol) for 5 min followed by washing three times using 100 μL PBS to remove the dye. A total of 100 μL of monosodium phosphate buffer (0.1M NaH_2_PO_4_ in 50% (*w*/*v*) ethanol) was added to wells at room temperature and shaken for 5 min for dye solubilization. Absorbance at 490 nm was used for LDH release or cell viability assay, whereas absorbance at 570 nm was used for the proliferation assay using a Bio-Tek microplate reader (Winooski, VT, USA). The results of LDH/Crystal violet are presented as a percentage change of viability/proliferation compared to the vehicle control.

### 4.3. Migration Study

During the development of atherosclerosis, the recruitment of monocytes to the affected area is a vital step underlying lesion formation. Therefore, we analyzed myricetin’s effect on the recruitment of THP-1 monocytes, and a migration assay was performed as previously described [43]. The monocyte migration in the presence or absence of myricetin was expressed as a percentage increase or decrease compared to the control.

### 4.4. Cells Treatment for Microarray Experiment

THP-1 cells were seeded at 1 × 10^6^ cells/well and were cultured in a 6-well plate with total volumes of 3 mL of RPMI complete medium and differentiated into macrophages by incubation with PMA (160 nM) for 24 h. These macrophages were treated with myricetin (25 μM) for an extra 24 h, while vehicle control cells were treated with DMSO (1%). Cells were lysed, and total mRNA was isolated using the Qiagen RNeasy Mini Kit (Qiagen, Hilden, Germany) for microarray experiments. SYBRGreen master mix (Qiagen, Hilden, Germany) and the ImProm-ii Reverse Transcription System (Promega, WI, USA) were used for double-stranded cDNA synthesis from macrophages.

### 4.5. Array Processing

GeneChip™ Human Gene 1.0 ST Array, GeneChip™ WT Terminal Labeling Kit, GeneChip™ Hybridization, Wash, and Stain Kit from Affymetrix (Affymetrix, Santa Clara, CA, USA) were used for transcriptional expression profiling. An in vitro transcription reaction was carried out to produce biotin-labeled cRNA, and then the target was fragmented. The cocktail of the fragmented target, biotinylated cRNA, control oligonucleotide B2, 20X eukaryotic hybridization control, 2X hybridization mix, DMSO, and nuclease-free water were hybridized with the GeneChip probe array at 45 °C and 60 rpm for a 16-h incubation period. The array was designed using human genome hg 18 with a set of 764,885 probes and 28,869 genes to profile the whole genome expression. The Affymetrix**^®^** GeneChip**^®^** Scanner 3000 was used for scanning images with .dat or .cel file extension. GeneChip operating software**^®^** version 1.2 (Affymetrix cat. no. 690036) was used to define experimental information such as probe cells, probe array type, sample description, and computing intensity for each cell.

### 4.6. Gene Expression Analysis

Partek**^®^** Genomics Suite (GS) 7.0 was used to analyze raw Affymetrix CEL files by calculating the probe intensity for myricetin-treated vs. the vehicle (control) group of macrophages. A robust multi-chip average program was applied for the normalization of a group of .cel files simultaneously. Principal component analysis (PCA) was performed to reduce the dimensionality of the dataset while preserving the variability. Differentially expressed genes (DEGs) were identified using ANOVA and a cut-off of false-discovery rate (FDR) with adjusted *p*-value ≤ 0.05 and fold change ≥ 2. Unsupervised hierarchical clustering was used to assess the overall expression pattern in treated (case) and untreated (control) cell lines.

### 4.7. Gene Ontology and Molecular Pathway Analysis

For further analysis of significant DEGs, gene set analysis or gene ontology analysis was carried out using Partek GS to annotate the dataset as the biological process, cellular component, and molecular function categories. Ingenuity Pathway Analysis software (Ingenuity Systems, Redwood City, CA, USA) was used to find significant canonical pathways, biological networks, biological functions, and phenotypes/diseases associated with the present study. DEGs, along with their corresponding Affymetrix probe sets ID/gene symbol/Entrez gene ID as clone identifier, *p*-value, and fold change values, were uploaded into IPA for functional analysis. The percentage and number of uploaded genes/molecules matching genes of a canonical pathway were measured as z-score, ratio, or Fisher’s exact test for significance. The molecule activity predictor was employed to predict the activation or suppression effects of a gene/molecule on other molecules of the pathway.

### 4.8. Statistical Analysis 

The IBM-SPSS program version 25 was used for statistical analysis. One-way ANOVA and Tukey’s post hoc analysis were carried out to compare more than two groups, and if only two groups were compared, *t*-test independent samples were used. The findings of three independent experiments are expressed as mean ± standard error of the mean (SEM), and *p*-value ≤ 0.05 was considered statistically significant [44].

## 5. Conclusions

Myricetin is safe and may be considered a preventative pharmaceutical for atherosclerosis and cardiovascular diseases. The THP-1 cell models are commonly used for mimicking the function and regulation of monocytes and macrophages in the vasculature. Although the THP-1 response can suggest possible ex vivo or in vivo responses, validation by in vivo investigations are must to reach more conclusive conclusions. The in vitro viability and proliferation assay result showed “no cytotoxicity” of myricetin on THP-1 macrophages. The significantly expressed and atherosclerosis-associated genes (*MSR1*, *CD36*, *NR1H3*) were detected by transcriptome analysis. Further, in silico prediction results showed a strong association of activation in upstream regulators and molecular pathways (LXR/RXR activation, phagocytosis macrophages) with atherosclerosis. We plan to further carry out in vitro/in vivo studies using an appropriate animal model as a continuation of this work. 

## Figures and Tables

**Table 1:** List of the top significantly expressed up and down-regulated genes.

Gene_Assignment	Gene_Symbol	*p*-Value	Fold-Change
NM_002445//macrophage scavenger receptor 1//8p22	*MSR1*	0.044	8.80
NM_000072//CD36 molecule (thrombospondin receptor)//7q11.2	*CD36*	0.003	8.30
NM_001293298//cell migration inducing protein, hyaluronan binding//15q24	*CEMIP*	0.036	7.01
NM_001130101//nuclear receptor subfamily 1, group H, member 3//11p11.2 //	*NR1H3*	0.024	6.67
NM_001001924//microtubule associated tumor suppressor 1//8p22	*MTUS1*	0.001	6.35
NM_145244//DNA-damage-inducible transcript 4-like//4q24	*DDIT4L*	0.005	4.78
NM_001130455//dysferlin//2p13.3	*DYSF*	0.049	4.31
NM_001306129//fibronectin 1//2q34	*FN1*	0.016	3.98
NM_001304441//matrix metallopeptidase 8//11q22.3	*MMP8*	0.021	3.97
NM_001444//fatty acid binding protein 5//8q21.13	*FABP5*	0.027	3.91
NM_001122//perilipin 2//9p22.1	*PLIN2*	0.019	3.79
NM_000582//secreted phosphoprotein 1//4q22.1	*SPP1*	0.042	3.71
BT007449//fatty acid binding protein 5 (psoriasis-associated)//8q21.13 //	*FABP5*	0.030	3.46
NM_006207//platelet-derived growth factor receptor-like//8p22-p21.3	*PDGFRL*	0.007	3.46
NM_001286449//TEX9//testis expressed 9//15q21.3	*TEX9*	0.004	3.44
NR_002324//small nucleolar RNA, H/ACA box 62//3p22.1	*SNORA62*	0.031	−3.27
NM_001136219//Fc fragment of IgG, low affinity IIa, receptor (CD32)//1q23	*FCGR2A*	0.037	−3.18
NM_014278//heat shock 70kDa protein 4-like//4q28	*HSPA4L*	0.011	−2.58
NM_001271594//SESN3//sestrin 3//11q21	*SESN3*	0.026	−2.27
NR_002590//SNORA41//small nucleolar RNA, H/ACA box 41//2q33	*SNORA41*	0.001	−2.20

**Table 2:** Top 10 canonical pathways revealed by Ingenuity pathway analysis.

Ingenuity Canonical Pathways	−log(*p*-Value)	Z-Score	Genes
LXR/RXR Activation	4.62	2.3	*CD36*, *MSR1*, *NR1H3*
VDR/RXR Activation	4.46	2.13	*IGFBP3*, *PRKCH*, *SPP1*, *TGFB2*
Inhibition of Angiogenesis by TSP1	3.71	2.21	*CD36*, *SDC2*
Phagosome Formation	3.69	2.121	*C5AR2*, *CD209*, *CD36*, *FCGR2A*, *FN1*, *ITGA3*, *MSR1*, *PRKCH*
Tumor Microenvironment Pathway	3.08	2.03	*FN1*, *MMP8*, *SPP1*, *TGFB2*
Cardiac Hypertrophy Signaling (Enhanced)	3.05	2.236	*ADCY8*, *ITGA3*, *PRKCH*, *RCAN1*, *TGFB2*
Hepatic Cholestasis	2.97	2.107	*ADCY8*, *NR1H3*, *PRKCH*, *TGFB2*
RAR Activation	2.87	2.06	*ADCY8*, *IGFBP3*, *PRKCH*, *TGFB2*
Role of NFAT in Cardiac Hypertrophy	2.73	2.04	*ADCY8*, *PRKCH*, *RCAN1*, *TGFB2*
Wound Healing Signaling Pathway	2.53	2.008	*FN1*, *ITGA3*, *MMP8*, *TGFB2*

## Data Availability

The expression data have been submitted to the NCBI’s GEO database with accession number GSE160430 (https://www.ncbi.nlm.nih.gov/geo/query/acc.cgi?acc=GSE160430 accessed on 20 June 2022).

## Supplementary Materials

The following supporting information can be downloaded at: https://www.mdpi.com/article/10.3390/ijms24010278/s1.

## References

[1] Williams J.W., Giannarelli C., Rahman A., Randolph G.J., Kovacic J.C. (2018). Macrophage Biology, Classification, and Phenotype in Cardiovascular Disease: JACC Macrophage in CVD Series (Part 1). J. Am. Coll. Cardiol..

[2] Luque M.C.A., Galuppo M.K., Capelli-Peixoto J., Stolf B.S. (2018). CD100 Effects in Macrophages and Its Roles in Atherosclerosis. Front. Cardiovasc. Med..

[3] Barrett T.J. (2020). Macrophages in Atherosclerosis Regression. Arterioscler. Thromb. Vasc. Biol..

[4] Nascimento C.R., Rodrigues Fernandes N.A., Gonzalez Maldonado L.A., Rossa Junior C. (2022). Comparison of monocytic cell lines U937 and THP-1 as macrophage models for in vitro studies. Biochem. Biophys. Rep..

[5] Chanput W., Mes J.J., Wichers H.J. (2014). THP-1 cell line: An in vitro cell model for immune modulation approach. Int. Immunopharmacol..

[6] Mishra T.K., Routray S. (2003). Current perspectives on statins. J. Indian Med. Assoc..

[7] Salvamani S., Gunasekaran B., Shaharuddin N.A., Ahmad S.A., Shukor M.Y. (2014). Antiartherosclerotic Effects of Plant Flavonoids. BioMed Res. Int..

[8] Kleemann R., Verschuren L., Morrison M., Zadelaar S., van Erk M.J., Wielinga P.Y., Kooistra T. (2011). Anti-inflammatory, anti-proliferative and anti-atherosclerotic effects of quercetin in human in vitro and in vivo models. Atherosclerosis.

[9] Andarwulan N., Batari R., Sandrasari D.A., Bolling B., Wijaya H. (2010). Flavonoid content and antioxidant activity of vegetables from Indonesia. Food Chem..

[10] Ross J.A., Kasum C.M. (2002). Dietary Flavonoids: Bioavailability, Metabolic Effects, and Safety. Annu. Rev. Nutr..

[11] Moss J.W.E., Williams J.O., Ramji D.P. (2018). Nutraceuticals as therapeutic agents for atherosclerosis. Biochim. Et Biophys. Acta Mol. Basis Dis..

[12] Semwal D.K., Semwal R.B., Combrinck S., Viljoen A. (2016). Myricetin: A Dietary Molecule with Diverse Biological Activities. Nutrients.

[13] Mulvihill E.E., Burke A.C., Huff M.W. (2016). Citrus Flavonoids as Regulators of Lipoprotein Metabolism and Atherosclerosis. Annu. Rev. Nutr..

[14] Wang Z.H., Ah Kang K., Zhang R., Piao M.J., Jo S.H., Kim J.S., Kang S.S., Lee J.S., Park D.H., Hyun J.W. (2010). Myricetin suppresses oxidative stress-induced cell damage via both direct and indirect antioxidant action. Environ. Toxicol. Pharmacol..

[15] Bhatia G., Khanna A.K., Sonkar R., Mishra S.K., Srivastava S., Lakshmi V. (2011). Lipid lowering and antioxidant activity of flavones in triton treated hyperlipidemic rats. Med. Chem. Res..

[16] Cho B.O., Yin H.H., Park S.H., Byun E.B., Ha H.Y., Jang S.I. (2016). Anti-inflammatory activity of myricetin from Diospyros lotus through suppression of NF-κB and STAT1 activation and Nrf2-mediated HO-1 induction in lipopolysaccharide-stimulated RAW264.7 macrophages. Biosci. Biotechnol. Biochem..

[17] Meng Z., Wang M., Xing J., Liu Y., Li H. (2019). Myricetin ameliorates atherosclerosis in the low-density-lipoprotein receptor knockout mice by suppression of cholesterol accumulation in macrophage foam cells. Nutr. Metab..

[18] Hou W., Hu S., Su Z., Wang Q., Meng G., Guo T., Zhang J., Gao P. (2018). Myricetin attenuates LPS-induced inflammation in RAW 264.7 macrophages and mouse models. Future Med. Chem..

[19] Hannon D.B., Thompson J.T., Khoo C., Juturu V., Vanden Heuvel J.P. (2016). Effects of cranberry extracts on gene expression in THP-1 cells. Food Sci. Nutr..

[20] Agraharam G., Girigoswami A., Girigoswami K. (2022). Myricetin: A Multifunctional Flavonol in Biomedicine. Curr. Pharmacol. Rep..

[21] Kim J.D., Liu L., Guo W., Meydani M. (2006). Chemical structure of flavonols in relation to modulation of angiogenesis and immune-endothelial cell adhesion. J. Nutr. Biochem..

[22] Ignatova I.D., Angdisen J., Moran E., Schulman I.G. (2013). Differential Regulation of Gene Expression by LXRs in Response to Macrophage Cholesterol Loading. Mol. Endocrinol..

[23] Remmerie A., Scott C.L. (2018). Macrophages and lipid metabolism. Cell. Immunol..

[24] Saenz J., Santa-María C., Reyes-Quiroz M.E., Geniz I., Jiménez J., Sobrino F., Alba G. (2018). Grapefruit Flavonoid Naringenin Regulates the Expression of LXRα in THP-1 Macrophages by Modulating AMP-Activated Protein Kinase. Mol. Pharm..

[25] Cuthbert G.A., Shaik F., Harrison M.A., Ponnambalam S., Homer-Vanniasinkam S. (2020). Scavenger Receptors as Biomarkers and Therapeutic Targets in Cardiovascular Disease. Cells.

[26] Park Y.M. (2014). CD36, a scavenger receptor implicated in atherosclerosis. Exp. Mol. Med..

[27] Freeman M.W. (1997). Scavenger receptors in atherosclerosis. Curr. Opin. Hematol..

[28] Jiménez B., Volpert O.V., Crawford S.E., Febbraio M., Silverstein R.L., Bouck N. (2000). Signals leading to apoptosis-dependent inhibition of neovascularization by thrombospondin-1. Nat. Med..

[29] Pennathur S., Pasichnyk K., Bahrami N.M., Zeng L., Febbraio M., Yamaguchi I., Okamura D.M. (2015). The macrophage phagocytic receptor CD36 promotes fibrogenic pathways on removal of apoptotic cells during chronic kidney injury. Am. J. Pathol..

[30] Zhao L., Varghese Z., Moorhead J.F., Chen Y., Ruan X.Z. (2018). CD36 and lipid metabolism in the evolution of atherosclerosis. Br. Med. Bull..

[31] Bernal-Lopez R.M., Llorente-Cortes V., López-Carmona D., Mayas D.M., Gomez-Huelgas R., Tinahones F.J., Badimon L. (2011). Modulation of human monocyte CD36 by type 2 diabetes mellitus and other atherosclerotic risk factors. Eur. J. Clin. Investig..

[32] Lopez-Carmona M.D., Plaza-Seron M.C., Vargas-Candela A., Tinahones F.J., Gomez-Huelgas R., Bernal-Lopez M.R. (2017). CD36 overexpression: A possible etiopathogenic mechanism of atherosclerosis in patients with prediabetes and diabetes. Diabetol. Metab. Syndr..

[33] Huh H.Y., Pearce S.F., Yesner L.M., Schindler J.L., Silverstein R.L. (1996). Regulated expression of CD36 during monocyte-to-macrophage differentiation: Potential role of CD36 in foam cell formation. Blood.

[34] Seimon T.A., Obstfeld A., Moore K.J., Golenbock D.T., Tabas I. (2006). Combinatorial pattern recognition receptor signaling alters the balance of life and death in macrophages. Proc. Natl. Acad. Sci. USA.

[35] Seimon T., Tabas I. (2009). Mechanisms and consequences of macrophage apoptosis in atherosclerosis. J. Lipid Res..

[36] Lian T.W., Wang L., Lo Y.H., Huang I.J., Wu M.J. (2008). Fisetin, morin and myricetin attenuate CD36 expression and oxLDL uptake in U937-derived macrophages. Biochim. Et Biophys. Acta.

[37] Kim G.D. (2017). Myricetin Inhibits Angiogenesis by Inducing Apoptosis and Suppressing PI3K/Akt/mTOR Signaling in Endothelial Cells. J. Cancer Prev..

[38] Cai Y., Yao H., Sun Z., Wang Y., Zhao Y., Wang Z., Li L. (2021). Role of NFAT in the Progression of Diabetic Atherosclerosis. Front. Cardiovasc. Med..

[39] Molkentin J.D. (2004). Calcineurin–NFAT signaling regulates the cardiac hypertrophic response in coordination with the MAPKs. Cardiovasc. Res..

[40] Fortelny N., Overall C.M., Pavlidis P., Freue G.V.C. (2017). Can we predict protein from mRNA levels?. Nature.

[41] Guo Y., Xiao P., Lei S., Deng F., Xiao G.G., Liu Y., Chen X., Li L., Wu S., Chen Y. (2008). How is mRNA expression predictive for protein expression? A correlation study on human circulating monocytes. Acta Biochim. Et Biophys. Sin..

[42] Perl K., Ushakov K., Pozniak Y., Yizhar-Barnea O., Bhonker Y., Shivatzki S., Geiger T., Avraham K.B., Shamir R. (2017). Reduced changes in protein compared to mRNA levels across non-proliferating tissues. BMC Genom..

[43] Almassabi R.F., Huwait E.A., Almowallad S.J., Saddeek S.Y., Gauthaman K. (2021). In Vitro: The Modulating Effect of Myricetin on the Atherosclerosis Related Processes in THP1 Macrophages. J. Pharm. Res. Int..

[44] Moorthy A., Venugopal D.C., Shyamsundar V., Madhavan Y., Ravindran S., Kuppuloganathan M., Krishnamurthy A., Sankarapandian S., Ganapathy V., Ramshankar V. (2022). Identification of EGFR as a Biomarker in Saliva and Buccal Cells from Oral Submucous Fibrosis Patients-A Baseline Study. Diagnostics.

