# Supplementary figures and images for: Microarray Expression Profile of Myricetin-Treated THP-1 Macrophages Exhibits Alterations in Atherosclerosis-Related Regulator Molecules and LXR/RXR Pathway
Source: Int J Mol Sci. 2022 Dec 23;24(1):278. doi: 10.3390/ijms24010278 (PMC9820668; doi:10.3390/ijms24010278)

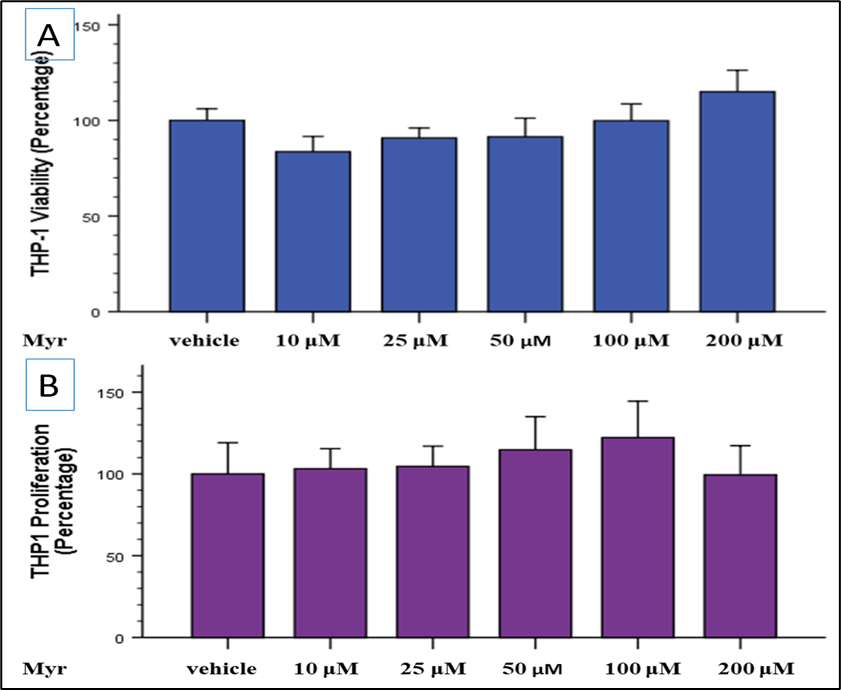

Supplement: Supplementary file 1 [file ijms-24-00278-s001.zip › Suppl Fig 1 to 3/Suppl FigS1 effect of myricetin on THP-1 macrophages.tif]

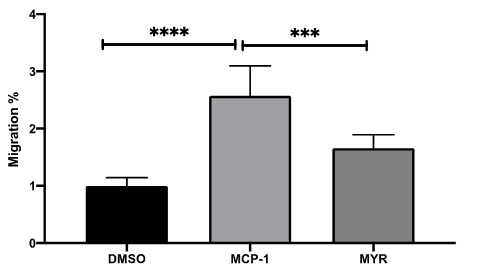

Supplement: Supplementary file 1 [file ijms-24-00278-s001.zip › Suppl Fig 1 to 3/Suppl FigS2 migration study 300.tif]

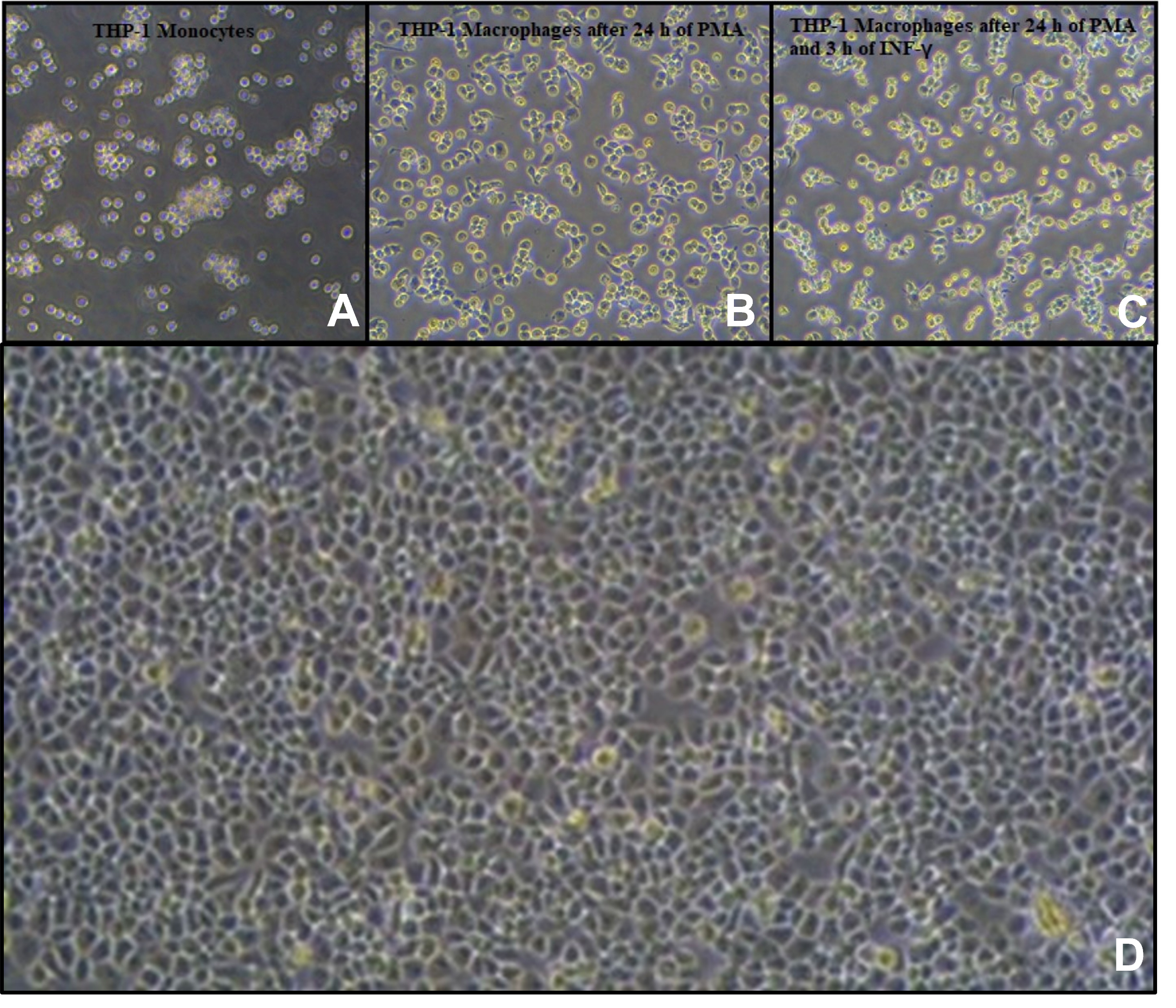

Supplement: Supplementary file 1 [file ijms-24-00278-s001.zip › Suppl Fig 1 to 3/Suppl FigS3 cell images.png]
